# Increasing research capacity with ICES Data & Analytic Services (DAS)

**DOI:** 10.23889/ijpds.v1i1.331

**Published:** 2017-04-19

**Authors:** Lisa Ishiguro, Refik Saskin, J. Charles Victor

**Affiliations:** 1 Institute for Clinical Evaluative Sciences (ICES)

## Background

The Institute for Clinical Evaluative Sciences (ICES) is a not-for-profit organization that conducts research to evaluate health care delivery and outcomes. Established in 1992, ICES houses a vast and secure array of linkable, coded health-related data on more than 13 million Ontarians, including health services data, health care provider data, registries and population-based health surveys. ICES has a reputation for generating strong evidence-based knowledge to inform policy and practice, however the use of the data was restricted to ICES' purposes. In March 2014, ICES launched the Data & Analytic Services (DAS) platform with the primary objective of increasing access to its data to publicly-funded researchers, health care providers and administrators, policymakers and students.

## Method

DAS provides access to highly de-identified, risk-reduced datasets created from ICES' data holdings; analytic support; and complete data analysis and report writing services. DAS also enables the importation of external data for linkage to ICES' data holdings. Research objectives and methodology are led by the requestor and ICES analysts rely on their subject matter expertise to direct the deliverables.

## Results

Since launch, over 200 requests from Canada, United States and United Kingdom have been adjudicated, of which 187 have been deemed feasible and eligible. Over the same period of time, ICES as an organization had over 700 active projects of which 348 were initiated, an increase in capacity of 26%. Though Toronto-based researchers represent the majority of the requests (62%), there have been requests from outside Ontario interested in comparing aspects of Ontario's healthcare to their home province. Research topics have varied and include assessments of health care provision by sector, disease prevalence and treatment, and statistical methods. An unexpected outcome of increasing access has been the large interest from small physician groups and knowledge users who are not typically involved in research for academic purposes. Access to ICES' data holdings provides an opportunity to examine a larger cohort of patients who share the same characteristics as their clinic patients or group. Furthermore, by enabling remote access to the data, DAS is able to leverage the capabilities of ICES' data holdings and increase research capacity in a short period of time.

## Conclusion

In making one of the most comprehensively linked health administrative data repositories in the world widely available to the broader research and healthcare community, DAS engages investigators involved in front-line care, stimulates new avenues of research and fosters collaboration that was previously unachievable.

